# Fabrication of a turbid optofluidic phantom device with tunable *μ*_*a*_ and *μ*′_*s*_ to simulate cutaneous vascular perfusion

**DOI:** 10.1038/srep30567

**Published:** 2016-07-26

**Authors:** Chen Chen, Midhat Ahmed, Tom Häfner, Florian Klämpfl, Florian Stelzle, Michael Schmidt

**Affiliations:** 1 Chair of Photonic Technologies, Friedrich-Alexander Universität Erlangen-Nürnberg, Konrad-Zuse-Str. 3/5, D-91052 Erlangen, Germany; 2 Erlangen Graduate School in Advanced Optical Technologies, Paul-Gordan-Str. 6, D-91052 Erlangen, Germany; 3 Department of Oral and Maxillofacial Surgery, Friedrich-Alexander Universität Erlangen-Nürnberg, Glückstr. 11, D-91054 Erlangen, Germany

## Abstract

Microfluidic devices are oftenly used to calibrate the imaging reconstruction, because they simulate the morphology of microvasculature. However, for lack of optical properties in microfluidics, the functional recovery of oximetry information cannot be verified. In this work, we describe the fabrication of a novel turbid optofluidic tissue phantom. It is designed to mimic the vascular perfusion and the turbid nature of cutaneous tissue. This phantom contains an interior hollow microfluidic structure with a diameter of *ϕ*_*ave*_ = 50 μm. The microfluidic structure includes the geometry of an inlet, a river-like assay and an outlet. This structure can be perfused by hemoglobin solution to mimic the cutaneous micro-circulation. The multiple-layered phantom matrices exhibit the representative optical parameters of human skin cutis, namely the absorption coefficient *μ*_*a*_ and the reduced scattering coefficient 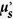. The geometry of the generated microfluidic structure is investigated by using Spectral-Domain Optical Coherence Tomography. This optofluidic phantom bridges the gap between tissue equivalent phantoms and Lab-On-Chip devices. Perspectively, this device can be used to calibrate a variety of optical angiographic imaging approaches.

Abnormal skin vascularization appears around a skin lesion, whereby the superficial vascular becomes densed and has inadequate blood perfusion. For vascular skin cancer or melanoma, the superficial vasculature and the surrounding extracellular matrix are provided with low oxygenation blood. Margin with such an abnormal skin vascularization is a common target to be removed during e.g. a Mohs surgery. In the diagnostic procedure of locating such a margin, a number of anatomic imaging techniques have been proposed. These technologies produce cross-sectional or projection images of the vascularization in cutaneous tissues, including scar tissue, granulation tissue, mucous cysts, leukoplakia and cancer neoplasm. The latest popularity is to investigate multi-modal imaging approaches to provide information about flow dynamics or oximetry of vascular perfusion during an anatomic imaging. optical angiographic imaging is better suited for this purpose. Several new optical techniques have been developed to recover the vascular perfusion in skin. These techniques include *Photon Acoustic Tomography* (PAT), *Optical Coherence Tomography* (OCT), *Laminar Optical Tomography* (LOT) and laser speckle imaging^1^,^2^,^3^. Some of these techniques are now reaching the clinical application stages.

As mentioned above, angiographic imaging approaches are gradually integrated to simultaneously give an architectural reconstruction and functional imaging of the blood perfusion. For the research on all techniques mentioned above, phantom validation is an essential strategy to provide clinically relevant insights into the device performance^4^,^5^,^6^,^7^. However, the established tissue phantom has not evolved into a multi-functional tool to calibrate the imaging reconstruction of multi-modal approaches. For instance, using a transparent microfluidics as the phantom for sOCT imaging brings critical points on the discussion about experimental results. The transparent microfluidics does not provide any turbidity like human tissue. Therefore, it is impossible to achieve an extra light reflectivity, like it is induced by the scattering in a real tissue. Consequently, a separation of absorbing and scattering factors of tissue must be re-done, when work is conducted from phantom validation onto e.g. an *in*-*vivo* study.

There are two established ways to fabricate such a multi-functional phantom: the traditional optical tissue phantom and the Lab-On-Chip microfluidic devices. The traditional phantoms can demonstrate the optical properties of skin cutis properly. Some researchers have successfully generated vessel-like structures in phantoms^8^. In the work of Bykov *et al.*, they present a solid-state phantom with a deposited capillary network. This phantom is constructed of polyvinyl chloride-plastisol with added titanium dioxide nanoparticles. Multi-layers are designed as a combination of layers with different thicknesses (0.3–1). Optical parameters are tailored to mimic those of pig-ear skin. A capillary network (*ϕ* = 0.2, 0.4, 0.8) resembling upper blood plexus is folded between different layers^9^. However, the dimension of the generated vessel is far away from the diameter of a real cutaneous microvasculature (0.01–0.1 mm). Obviously, there is a limit to the ability of traditional phantoms regarding mimicking the real dimension of the microvasculature.

Microfluidic devices provide a Lab-On-Chip solution for imitating the micro-circulation. With the assistance of microfluidics, optical imaging systems are calibrated for extracting hemodynamic information from biological tissue samples. In the review work of Wu *et al.*^10^, a full set of application cases of using microfluidic devices to evaluate the performance of the optical imaging techniques are provided. The applications involve microscopes, interferometer-based systems, digital inline holography and scanning-based imaging devices. Materials that can be downscaled to the chip dimension include membrane^11^, *Thermoset Polyester* (TPE), *Polyurethane Methacrylate* (PUMA) and *Norland Adhesive 81* (NOA81). Using laser micro-machining to fabricate a dynamic flow microfluidic phantom, Luu *et al.* show that even more common materials could be used for the fast prototyping of the micro-channels (*ϕ* < 20 μm). These materials include epoxies, plastics and household tape^12^. Among all the materials, PDMS is the most common choice as the matrix of microfluidic devices. It makes fabrication easy and is compatible with many bio-assays. Raquin *et al.* describe the fabrication of a multiple-layered PDMS device, which consists of two identical non-communicating capillary networks. Each network contains staggered channels in 2 mm deep and 0.9 mm wide^13^.

A unique example comes from Parthasarathy *et al.* In their work, microfluidic devices are designed and fabricated using PDMS to mimic the capillary network in brain. The microfluidic structure has a dimension in a range of *ϕ* = 10–150 μm. This microfluidic device is used as a tissue phantom to investigate the influencing factors on the contrast of laser speckle imaging^14^. Another contribution of this work is that it proves the possibility of mixing PDMS and *Titanium Oxide* (*TiO*_2_) particles to achieve a certain level of scattering in the matrix. The maximum concentration of *TiO*_2_ reached is about 1.8 per gram of PDMS. This concentration does not disturb the hydrophilic or hydrophobic properties of PDMS. But at this concentration, the scattering level of a real epidermis cannot be achieved. The reason is that PDMS, also similar for other materials mentioned above, needs a chemical clearance to make a change in hydrophobicity to seal e.g. onto the glass wafer. This necessity increases the difficulties of assigning the chip appropriate scatter or absorber, because an insertion of anything (such as *TiO*_2_) will disturb the cross-linking at the boundary of the matrix material. Besides, methods like laser direct write brings changes in scatter (such as *TiO*_2_) with its optical properties.

Focusing on imaging the superficial cutaneous vasculature, a phantom device with turbid matrices and perfusable microfluidic vessels is needed. These two main features display respectively the optical properties of human skin cutis and the morphology of the microvascular perfusion. The most commonly referred anatomy of the skin vascularization is the two-layer-geometry. It describes the morphology, where a vascular network is folded between 2 thin slabs. A defined thickness is given to the slabs to mimic the epidermis and dermis respectively. The vascular network includes the arterio, the microvasculature and the venous (see Fig. 1).

This report introduces the fabrication of a cutaneous equivalent phantom with micron-scale interior hollow microfluidic structure to the two-layer-geometry. The technical objective is to fabricate a multiple-layered optofluidic device with turbid matrix. Decreasing the diameter of the embedded microfluidic structure to a value resembling a real microvasculature is one major challenge. Another challenge is to replicate the representative optical parameters *absorption coefficient μ*_*a*_ and *reduced scattering coefficient* 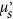 of skin cutis. These optical properties must not be significantly affected during the fabrication procedure. Regarding this, the fabrication process of neither the traditional optical phantoms nor the microfluidics offers us a compromising solution. In the following sections, we present our novel method, embedding-and-etching, to fabricate the turbid optofluidic Lab-On-Chip phantom. We also report the preliminary results from an OCT investigation of the microfluidic geometry. Meanwhile, spectroscopic characterization of the optical parameters of phantom matrix is presented.

## Results

### Investigating the vasculature geometry

**Figure 2:**
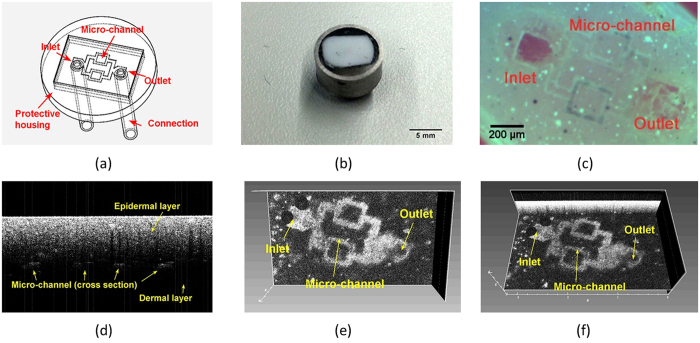
(**a**) Schematic of the generated vasculature in the finished sample (**b**) Photo of the finished prototype with two scattering layers (**c**) Photo of the micro-channel in phantom with one transparent layer (**d**) OCT B-Scan image of the cross section, which indicates the two-layer geometry (**e**) OCT top view of the generated microvasculature (**f**) OCT C-Scan 3D volume view of the generated microvasculature.

We prepare a prototype specimen containing the inlet, outlet and 4 separate rows of hollow micro-channels finished sample see Fig. 2, schematic and photo see Fig. 2(a,b and c). The diameter and the depth of the micro-channels are measured separately on the finished phantom using SD-OCT (Telesto-II-SP1, Thorlabs GmbH, Germany) within a 2-D B-Scan and a 3-D C-Scan. The microfluidic structure is injected with 4% intralipid solution to be highlighted as a white scattering target in the OCT image. Figure 2(d) shows the cross section of the sample, where a clear view of the two-layer-geometry is displayed. The flat fleckles represent the cross section of the generated micro-channels. Figure 2(e) demonstrates the top view of the hollow microfluidic structure. As shown in these images, the inlet and outlet mimic respectively the arterio and the venous, while the micro-channel represents the vasculature. Regarding a volume shrinkage ratio of the PU material, the estimated diameter of the channels should be around 55 μm. The measured value (from Fig. 2(e)) is 52 μm in average. This shows an excellent agreement to the estimated value. In order to validate the vessel geometry more directly, we refer to the OCT for a 3-D reconstruction (see Fig. 2(f)). The vessels are uniform in diameter along the entire structure. Overall, the reconstructed microfluidic structure shows no difference to the DFX-drawing. The geometric parameters of the generated microflduic structure conforms the intension of the design.

We install a diaphragm liquid dosing pump (SIMDOS, Carl Roth GmbH, Germany) as the alignment for the perfusion function. The dosing pump continuously injects meta-hemoglobin solution into the generated microfluidic structure to mimic the micro-circulation. The SIMDOS diaphragm liquid dosing pump gives a fast, precise feed of liquid into the defined flow rate from 1 to 100 mL/min. This fluidic dynamic simulates the vascular blood perfusion.

There are factors that may limit the size of the generated microfluidic structure. Firstly, most researchers working on angiographic imaging adopt microfluidics with a channel diameter of 50–100 *μ*m to examine the imaging resolution. Further decreasing the channel width to 20 *μ*m is technically possible. However, the success rate of producing a microfluidic structure in this dimension is lower, as indicated later during the discussion about the conformity rate. In this report, we demonstrate the fabrication procedure for 50 *μ*m diameter microfluidics, because it provides a best combination of production rate and technical specification to fulfill the need for replacing the microfluidic devices used/established. Also, most micro-vessels in the skin subsurface have a diameter of around 10–80 *μ*m. To reflect this anatomy, we choose the middle value of 50 *μ*m, which can actually be flexibly customized.

### Optical properties compared with *ex*-*vivo* skin

We expect significant similarities with a comparison of the optical properties of phantom matrix to those of the *ex*-*vivo* human cutis. Particularly, we characterize the representative parameters of *μ*_*a*_ and 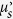 of the phantom matrix. The reference optical properties are reported elsewhere in refs 15, 16, 17. The paradigm of *μ*_*a*_ and 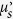 are attained from the skin slabs of *ex*-*vivo* human objects at the wavelengths of 700, 800, 900 and 1000 nm. Similarly, we measure the same coefficients from the PU slab at the same wavelengths by using a UV-IR spectrophotometer (UV-3600 series, SHIMADZU Co., Japan). All measured spectra are converted using a single integration sphere and *Inverse Adding Doubling* (IAD).

**Figure 3:**
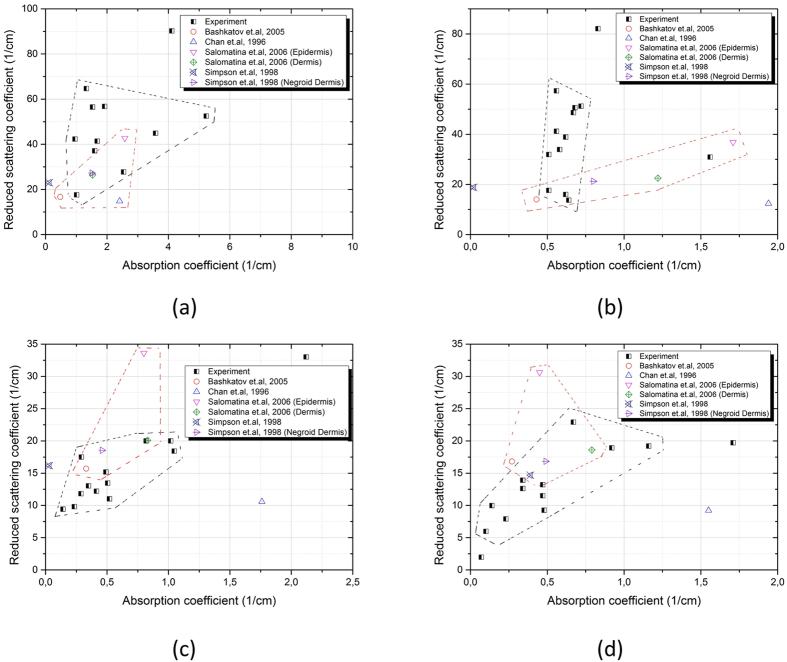
Optical properties of the phantom matrix at wavelengths of (**a**) 700 nm, (**b**) 800 nm, (**c**) 900 nm, (**d**) 1000 nm. The measured values are displayed in black dots and all locate in the sampling zone circled by black segmented lines. The paradigm zone conclude the optical properties of the real *ex*-*vivo* caucasian skin type. Reference points are located in paradigm zone circled by red segmented lines.

The results are found in Fig. 3. At the wavelength of 700, 900 and 1000, the *μ*_*a*_ and 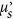 are close to the paradigm, where the sampling zone overlaps with the paradigm zone (as labelled in Fig. 3) However, the sampling zone at 800 does not cover the paradigm zone (as shown in Fig. 3(c)). This can be further modified by reducing the amount of *TiO*_2_. Nevertheless, this is not disturbing, when *μ*_*a*_ and 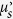 mostly approach the values of the *ex*-*vivo* human cutis. The points at the right section is regarded as an exception, which is not included in any circled zone. This point is sampled upon the high absorbing PU slab, which simulates the negroid epidermis.

### Characterizing the attenuation in optical properties

Using embedding-and-etching instead of e.g. laser direct write method^18^ to fabricate the microfluidic structure helps to avoid the scorch of *TiO*_2_ insertion in phantom matrix. However, the etching progress might alter the optical properties of the phantom matrix. Attenuation of the optical properties possibly stems from the contact of slab surface with the electrolyte. *NaHCO*_3_ is added into the electrolyte to change the PH values, so as to prevent the *CuCl*_2_ from condensing. A side effect by doing this is that a lower PH might induce the swelling of PU slabs. Due to this, we use the spectrophotometer to characterize the optical properties through the phantom matrix before and after etching. To verify the reproducibility of these results, same experiment are repeated on all slabs with different amount of *TiO*_2_ and India ink.

**Figure 4:**
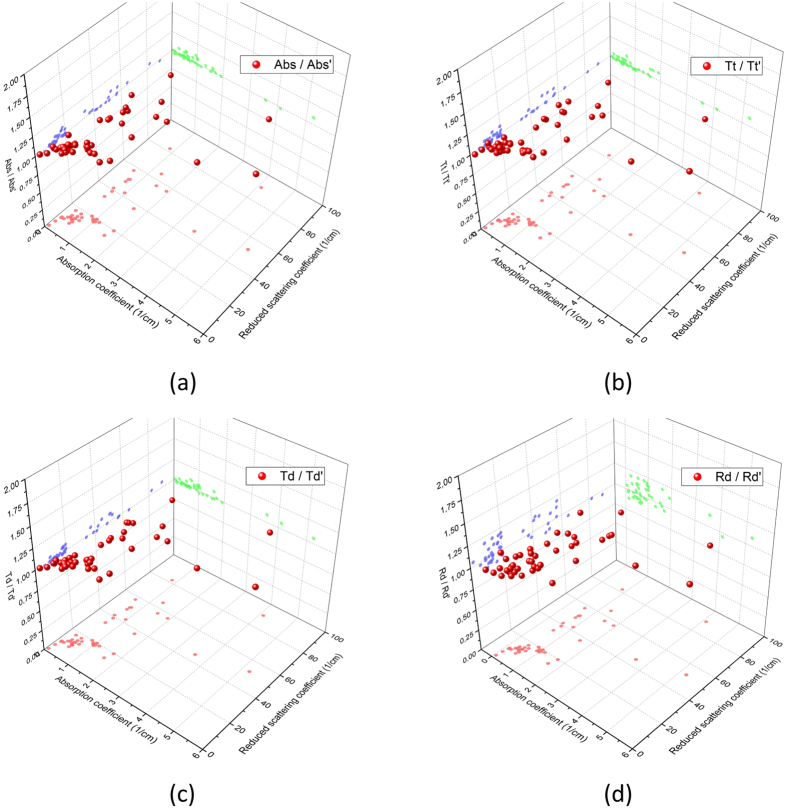
Optical properties of the PU slab before and after etching are characterized through the normalized spectra of (**a**) Absorbance, (**b**) Total transmittance, (**c**) Diffuse transmittance, (**d**) Diffuse reflectance.

Figure 4 summarizes the attenuation rate of the *total transmittance T*_*t*_, *diffuse transmittance T*_*d*_, *absorbance A*_*b*_ and *diffuse reflectance R*_*d*_ before and after etching. These parameters are correlated to the values *μ*_*a*_ and 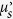. The Z-axis indicates the projection of the normalized value of e.g. *A*_*b*_. These values are supposed to be 1, as if the attenuation did not occur. The spectra of *A*_*b*_, *T*_*d*_ and *T*_*t*_ do not change with the etching progress. The registered values are around 1 in their correlation with the optical parameters *μ*_*a*_ and 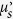. The standard deviations of these values do not exceed ±5%. The values for *R*_*d*_ alter more after etching. They range between 0.89 and 1.12. This change is mostly caused by the induced change of the surface status, as the *TiO*_2_ particles on surface of PU slabs is wetted by water molecules during etching. Despite this reason, the change could be also caused by the cross talk from the spectrophotometer, as the integration sphere in our alignment is limited in size. Therefore, this change is rated as rather slight and acceptable. By comparing the normalilzed *T*_*t*_, *T*_*d*_, *A*_*b*_ and *R*_*d*_, it can be verified that the electrolyte does not reveal any significant alteration in the optical parameters of the turbid PU slabs.

## Discussion

We have successfully fabricated the prototype optofluidic phantom. It contains an interior microvasculature including 4 separate perfusable micro-channels. The phantom matrix replicates the optical parameters *μ*_*a*_ and 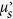 of human skin. To our best knowledge, our prototype is so far the only device, which simultaneously contains the microfluidic feature and the turbid nature. Most previous researches (including our work^19^) on phantom or microfluidics claim that the lamination of the mutiple layers of skin cutis can not be simulated^18^,^20^. In contrast, our prototype has multiple layers. Compared to the results from other literatures^21^,^22^, the diameter of the generated micro-vessels is closer to that of a real cutaneous vasculature. Unlike the established Lab-On-Chip devices, as described in literatures^18^,^20^, the optical properties of our prototype can be flexibly customized. Table 1 gives a brief comparison of the previous work and ours. By using our turbid optofluidic phantom, the impact from the turbid issue on the imaging reconstruction can be better comprehensively investigated. In our lab, we plan to reconstruct a 2-D velocity field of the hemoglobin flow via Doppler-mode OCT upon our phantom. Calibrating the flowmetry parameters of *velocity u* through the turbid media can be one of the perspectives.

Perspectively, the optofluidic phantom can be used to evaluate the performance of variant optical angiographic imaging approaches. Regarding spectrographic imaging, we would suggest e.g. an application in Laminar Optical Tomography to reconstruct oxygenation saturation changes of dermal lesions. In this case, a densed capillary network and a worse oxygenation of the injected hemoglobin solution mimic the cancerous dermal lesion. Other possible applications include calibrating spectroscopic OCT for recovering the distribution of *μ*_*a*_ and *μ*_*s*_ of skin tissue. It is also practicle to use Diffuse Reflection Imaging to recover the hemoglobin concentration *CtHbO*_2_ and *CtRHb* of hemoglobin solution in the optofluidic phantom^23^. Also, this optofluidic phantom can be used to evaluate the imaging resolution of PAT or LOT and the recovery precision of laser speckle imaging (of flow velocimetry) etc. In all these perspectives, the optofluidic phantom can assist us to directly understand how the turbid nature of skin affects the reconstruction precision.

### Delamination of layers

**Figure 5:**
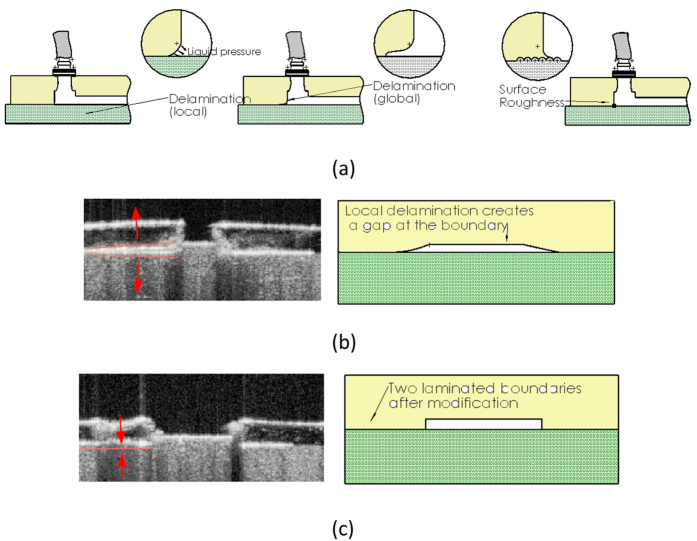
(**a**) A schematic of the delamination effect between the slabs and a modification method by changing the contact angle between PU material to prevent local delamination; OCT image and schematics of a partially transparent device which contains two layers (**b**) with delamination and (**c**) without delamination.

High pressure resistance is essential for all microfluidic devices. A strong, covalent bonding is needed in order to withstand high local pressures generated inside the flow. Weak bonding between layers leads to the delamination, which can result in a cross-contamination of fluids between the layers or disintegration of multiple layers (see Fig. 5 (a)). In our previous experiment, the stacking of PU slabs is done by simply casting PU upon another solidified PU slab. However, the depressed pit on the boundary can be teared under the lash of high-pressure (see Fig. 2a). To address this, the wetting possibility (contact angle) of the PU slab surface must be changed. Moreover, a larger effective contact area definitely helps to strengthen the bonding. In order to enlarge the contact area, a certain roughness is brought onto the surface of the slab. Experimentally, we rub the slab by using a fine-core sand paper (1200 grit). Also to view the delamination, we perform OCT experiments upon one of our phantom. This prototype has a transparent upper layer to give a highlighted reconstruction of the boundary under OCT (top view see Fig. 2b,c). Two samples are prepared: one with the boundary rubbed, while the other without any processing. We drill holes on random positions and inject water into these holes.

Figure 5(b) demonstrates a delamination at the boundary of 2 layers. This delamination is presented as a gap between 2 stacked layers. The delaminated boundaries are labelled as red lines near to them, with arrows pointing the direction of the delamination. On the other side, the layers are tightly attached to each other as shown in Fig. 5(c), after the slab surface is grounded before casting. This means, the effect of a grinding processing of the thin PU slab helps to maintain the stacked layers laminated. This strong lamination prevents the crash of the multiple-layered microfluidic phantom under a high local pressure.

### Homogeneity and selectivity in material

The homogeneity of scatters influences the light propagation in turbid media. An agglomeration of *TiO*_2_ may exist after the matrix material is solidified. This forms a scattering cluster. Some phantoms are constructed to contain a certain heterogeneity to mimic specific chromophores, such as hair follicle or sebaceous gland. Some researchers purposefully use heterogeneous phantom to test their imaging algorithms^24^. Nevertheless, most phantoms require a high homogeneity in matrix.

**Figure 6:**
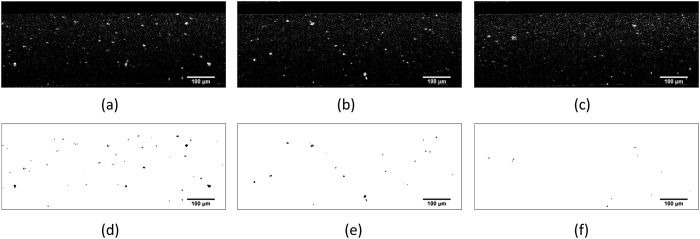
The original OCT B-Scan image of the cross section of (**a**) a typically inhomogeneous tissue phantom (**b**) more homogeneous tissue phantom, (**c**) best homogeneous tissue phantom subfigures; Subfigures (**d**–**f**) demonstrate the segmented scattering clusters corresponding to (**a**–**c**).

The scattering clusters can be reduced by a longer ultrasound bath. The ultrasound wave smashes and disperses the powder agglomeration. Experimentally, a 2 h ultrasound bath is executed after stirring. The homogenization process has been reported elsewhere^19^. To check the consequence of this homogenization, scatter homogeneity is evaluated from the OCT B-scan. The OCT images are de-speckled by filtering with high db value using an image post-processing software (ImageJ, National Institutes of Health, US). This image post-processing are performed on the OCT images, which represents before and after homogenization. The scattering clusters are thresholded and morphologically opened during the post-processing (see Fig. 6 from (a–c), or from (b–d)). By comparing Fig. 6(c,d), we find that the speckling spots are vanishing from Fig. 6(d). This shows that the scattering clusters are decreasing with the ultrasound bath.

**Figure 7:**
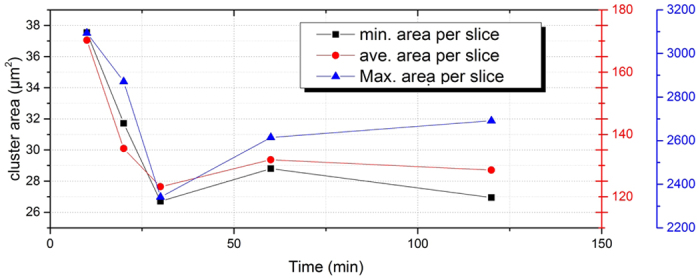
The maximum, minimum and average values of cluster area per slice OCT image correlated to the homogenization duration of the ultrasound bath.

Numerical quantification of the matrix homogeneity is implemented upon the thresholded OCT images. The maximum (*A*_max_), minimum *A*_min_, as well as the average values *A*_*ave*_ of the cluster area (per slice of OCT image) are calculated and correlated to the ultrasound bath duration (as shown in Fig. 7(g)). As indicated in figure, the *A*_max_ decreases from 3200 *μ*m^2^ (10 min sonification) to 2350 *μ*m^2^ (30 min sonification). However, a longer ultrasound bath does not help to further improve the homogeneity. Instead, the value slightly rebounds back to 2650 *μ*m^2^. For the *A*_min_ and *A*_*ave*_, a similar conclusions can be drawn. This means, a 30 min ultrasound bath is sufficient to distribute the *TiO*_2_ cluster into the PU mixture. An even longer sonification is not suggested, since the *TiO*_2_ particles start to come back into agglomeration. Researchers can define the homogenization based on the result, since different levels of homogeneity are required depending on different objectives.

The selection of matrix material is flexible. The applicable types of matrix materials include epoxy resin, agar gel, silicon gel, acrylic polymer, hemodialysis membrane etc. The selection of the matrix material is decided according to the experimental purposes. For instance, using polyurethane or epoxy resin helps to achieve a rigid mechanical property for a long-term stand. The silicon gel or acrylic polymer mimic the bio-mechanical elasticity of skin tissue. Phantom with defined elasticity can be used to calibrate elastographic imaging approaches, such as Ultrasound microscopy. To simulate a certain functional property of the real tissue, for example, the oxygenation consumption, hemodialysis membrane can be the option^25^. This simulates a hypoxia in cancer and can be used to calibrate e.g. PAT or sOCT. As long as the materials can be laminated with each other, they are regarded as suitable for the phantom matrix.

### Conformity rate and others

A optofluidic device for future commercial use requires a high conformity rate. The conformity rate of the optofluidic phantom is influenced by the dimension of the micro-channel; the recipe of the phantom slab etc. Table 2 shows the conformity rate of the optofluidic phantom correlated to these factors. The conformity is also graded at different process stages. In the feasibility study of preparing phantom slabs and copper mould, we repeat the experiments (approx. 150 times) to obtain enough pre-prerapred samples for later production. In the latest peroid of samll batch production, 10 prototype phantoms are prepared. The conformity rate is graded out of these 10 prototypes.

Some technical issues are noticed. They are labelled as circled numbers in Table 2. The reason and solution are presented as following. A conformity rate of 3 successes out of 10 attempts is acceptable.

- ① The minimum diameter achieved is 24 *μ*m. However, to produce a copper mould in this size is challenging. The entire drawing must be modified to compensate for the laser beam width (kerf).
- ② Air bubbles could be induced in the PU material during the preparation of the phantom slab. The air bubbles are classified into 2 types: mechanical air bubbles and chemical air bubbles. Mechanical air bubbles are created by stirring. They can be simply eliminated by using Ultra-sound bath instead of stirring. We use a vacuum autoclave to extract the air bubbles from the viscous PU mixture before casting. Chemical air bubbles come from the chemical reaction between PU and water molecules (which creates *CO*_2_). The water molecules are mostly imported by the ink. For this reason, it is noticed that negroid epidermis mimicking phantom slab has a lower rate of conformity. To solve this problem, casting is executed at 47 °C in a convection chamber (BINDER GmbH, Germany), where a dehydrated ambience helped to reduce the reactive air bubbles in the PU. Besides, we suggest to use epoxy resin to prepare the negroid mimicking slab.
- ③ Safty issue must be mentioned. *Cl*_2_ gas is produced during the etching progress. We suggest cover the electrolyte with a gas collecting jar, although chlorine also dissolves in water.
- ④ To improve the conformity rate, especially by embedding-and-etching, we suggest using other metal material with a higher chemical activity than copper. The electrolysis reaction can be then accelerated. Moreover, precise positioning of the drilling hole also simplify the perfusion of electrolyte into micro-channels. This also improves the conformity rate. For this, we suggest using a CNC micro-drilling machine for a precise positioning of drilling holes.

## Conclusion

In the framework of preparing a turbid optofluidic phantom for calibrating angiographic imaging of the cutaneous vasculature, a prototype with microfluidic structure is constructed. Our prototype is the only device, which simultaneously provides the alternatives in the microfluidic feature, the turbid nature, multiple layers and the perfusion function. All these features are merged into one device.

- Optical properties similar to real *ex*-*vivo* caucasian skin tissues are replicated and are not attenuated by the embedding-and-etching progress.
- Multiple layers are constructed to mimic the cutis, i.e. epidermis and dermis; delamination is avoided. The fabrication of the optofluidic phantom does require any specific material, like PDMS. This feature makes the choice of matrix material much wider and much less expensive.
- We confirm the feasibility of creating a vessel structure with a diameter of 52 μm at a depth of 150 to 200 μm from the phantom surface. The geometry is investigated using a Spectral Domain-OCT.

Future work will be conducted to upgrading the geometric complexity of the vascular pattern. We propose to generate a network of micro-channels to achieve the microfluidic nature; We shall adopt Agar gel as the matrix material, so as to simulate the clinical mechanical properties (elasticity) of the cutis; Application shall be performed to calibrate a variation of imaging reconstruction via OCT, diffuse reflection imaging etc. Similar experiments can be performed on Laminar Optical Tomography, Photon Acoustic Tomography, laser speckle imging etc.

## Methods

**Figure 8:**
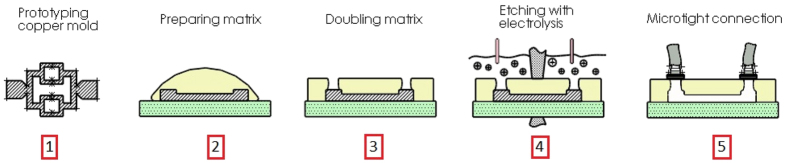
Schematic of the embedding-and-etching procedure, the layout from step 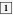 designing the mould and cut the copper mold using a ps-Laser, 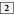 Preparing the PU matrix material, 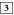 Stack the PU slabs with copper mould, 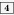 embedding-and-etching 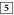 optofluidic phantom connected (e.g. with *MicroTight*^®^ tubing).

First, we draw the microfluidic structure on a copper film. Copper can be easily machined into a mould with a ps-laser. This structured copper mould is embedded between 2 *polyurethane* (PU) slabs. Polymer does not alter the chemical properties of copper. A contact between an electrolysis pool and the embedded copper is made by drilling holes at the phantom slab surface. The electrolyte perfuses through the hole and dissolves the embedded copper mould. After the copper mould is eliminated, hollow microfluidic structure is folded between the slabs. During the optical imaging experiments, the hollow structure can be perfused with a range of solutions, such as meta-hemoglobin solution or intralipid solution. Different PU slabs simulate the epidermis and dermis respectively to reflect their optical properties. In this case, *μ*_*a*_ and 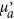 of the real *ex*-*vivo* human cutis are replicated. Figure 8 shows the embedding-and-etching procedure.

### Preparing the casting mould

Our aim is to simulate the superficial microvasculature that generally underlies the skin’s epithelial layer. The diameter of a cutaneous vasculature ranges from 10 to 80 μm. The vascular network is mostly located in the first 200 μm of skin. To simulate this, we intend to derive thin hollow micro-channels with a diameter *ϕ* = 50 μm at the depth of 150 to 200 μm from the surface. The microvasculature mimicking structure should achieve a certain geometric complexity. Therefore, we attempt to fabricate a river-like pattern, through which the blood flow from the artery through the capillary network into the vein is simulated.

**Figure 9:**
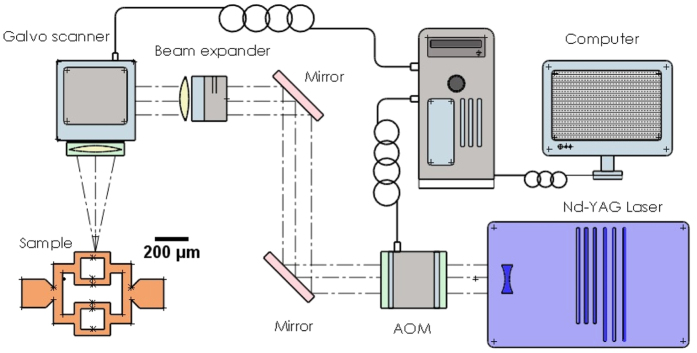
Schematic of the ps-laser setup and the light path (*P*_*p*_ = 25 mW; t = 10 ps; *F*_*p*_ = 200 KHz; *λ* = 532 nm).

In our design (as shown in Fig. 8), the inlet is split into 4 branches and then merge again to the outlet. The network pattern is designed using Solidworks (Dassault Systemes S.A., France). The inlet is located on the left and has a diameter of 1 mm to accommodate the incoming fluidic flow. The outlet in the same dimension is situated on the right-hand side on a symmetrical position. The diameter of the branches between inlet and outlet is set at 50 μm. We use a ps-laser (TBWP Fuego ps-laser, Time-Bandwidth Products AG, Switzerland) to cut this geometry from a copper film. To remove the metal by laser ablation, the beam spot is positioned and scans over the copper film surface according to the DFX-drawing from Solidworks (see Fig. 9). By this method, the copper mould with a premium edge quality is produced. In order to maintain the reproducibility of the channels’ dimensions of the copper mould, several laser parameters are kept constant. The output power of the laser is set at 1.45 W and beam diameter of 10 μm. The frequency of the pulse repetition rate is set to 25 kHz and the linear scanning speed is 600 mm/s.

### Preparing the matrix material

For the simulated studies on human cutis via optical imaging approaches, for example, with diffuse reflection imaging, the matrix should have a similar *μ*_*a*_ and 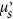 to that of a real skin tissue. Therefore, we choose the data from *ex*-*vivo* bio-metrologic studies^15^,^16^,^26^,^27^,^28^,^29^. The epidermis mimicking slab is prepared with a thickness of 200 μm, while the dermis mimicking slab is in 400 μm thickness. These fit the morphology of a real human skin. In the same way, it is simple to simulate other tissue types, such as retinal, mucosa etc. To do this, the thinkness, as well as the optical parameters should be customized.

The phantom matrix is constructed of castable aliphatic polyurethanes (WC-783, BJB Enterprise Co., US). To replicate the optical properties of skin, a certain amount of *TiO*_2_ powder (Sigma-Aldrich GmbH, Germany) and India ink (Pelikan GmbH, Germany) is inserted into the PU. The recipes, together with the mimicked skin types are listed in Table 3. The additives are dispersed by stirring for 30 min, followed by an ultrasound bath (Elmasonic P, Elma Hans Schmidbauer GmbH, Germany) at 45 °C. The solidified slab is de-moulded after 12 h, when the solidified slab becomes rigid enough for a long-term use. The dermal layer is directly casted upon the solidified epidermal layer, where the copper mould is positioned inbetween.

### Etching the retained copper wires

**Figure 10:**
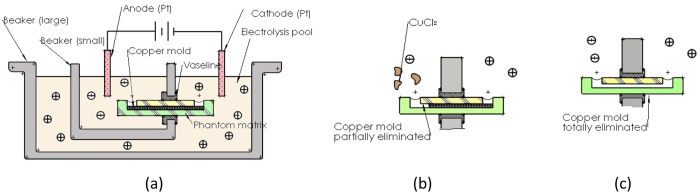
(**a**) Schematic of the electrolysis pool. (**b**,**c**) step by step explaination of the removal of the copper mould.

The folded copper mould is eliminated from the solidified phantom by an electrolysis reaction. The schematic of the etching pool is shown in Fig. 10. The pool consists of 2 plastic beakers. They are connected by the copper mould in phantom. This makes it possible for electrons to pass from one beaker to the other. Vaseline is used to stop the e-conduction other than that through the copper mold. We use the platinum wires as the anode/cathode pair. Over-saturated *sodium chloride* (NaCl) solution is prepared as the electrolyte. On the cathode, the copper captures the electrons from the NaCl and turns into *copper chloride* (*CuCl*_2_). The *CuCl*_2_ swims into the pool against the electron flow. On the anode, the NaCl captures the electrons and becomes *chloride gas* (*Cl*_2_).

To avoid the swelling of PU slabs, temperature is restricted to 23 degree celcius. We refresh the solution every 24 h to avoid material stagnation on the local etching knot. Moreover, *sodium bicarbonate* (*NaHCO*_3_) is inserted as an adjuvant. This adjusts the PH value of the electrolyte and prevents the *CuCl*_2_ from condensing. The electrolyte is pressed into the microfluidic structure and dissolves the copper mould without disrupting the walls of the channels. Thus producing the finished device. The entire etching procedure takes 48 h in average.

## Additional Information

**How to cite this article**: Chen, C. *et al.* Fabrication of a turbid optofluidic phantom device with tunable *µ_a_* and *µ_s_*’ to simulate cutaneous vascular perfusion. *Sci. Rep.* **6**, 30567; doi: 10.1038/srep30567 (2016).

## Figures and Tables

**Figure 1:**
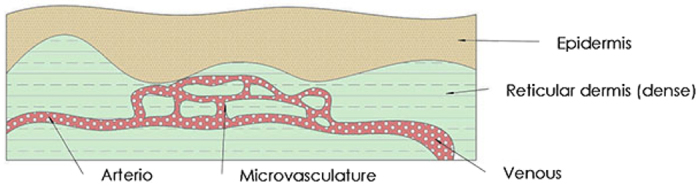
The anatomic structure of the superficial vascularization of cutaneous tissue with a two-layer-geometry.

**Table 1:** Comparison of the feature provided by the established phantom/microfluidic devices.

Feature/Group	Turbid matrix	Multiple layers	Perfusion function	Micron-scale structure
Wang *et al.*^21^	Yes	Yes	No	No
Sollier *et al.*^20^	No	No	Yes	Yes
Dabrowski *et al.*^30^	Yes	No	Yes	No
Samuel *et al.*^31^	No	Yes	No	Yes
Lim *et al.*^18^	No	No	Yes	Yes
Tuchin *et al.*^22^	Yes	Yes	Yes	No
Bykov *et al.*^9^	Yes	No	Yes	No
Bykov *et al.*^32^	Yes	Yes	Yes	No
Parthasarathy *et al.*^14^	limited	Limited	Yes	Yes
Chen *et al.*^19^	Yes	No	Yes	Limited

**Table 2:** Conformity rate correlated to the phantom recipe; dimension of the micro-channel and in different fabrication stages.

	Phantom recipe	Micro-channel dimension	Process niveau
Preparing copper mold ①		Dia. = 50 *μ*m: 17/20	
		Dia. = 20 *μ*m: 3/15	
Preparing PU slab ②	Caucasian: 73/100		
	Negroid: 3/20		
Embedding copper mold			8/10 (10 prototypes prepared)
Drilling holes			6/8 (8 from successful embedding)
Etching copper mold ③			3/6 (6 from successful drilling)
Connecting to pump			3/3 (3 from successful etching)
In total ④			3/10

**Table 3:** The phantom recipe; the concentration of *TiO*_2_ and india ink correlated to the mimicked skin type.

Phantom Nr.	Mimicked skin	Ct [*TiO*_2_] (g/40 mL PU)	Ct [Ink] (*μ*L/40 mL PU)	Mimicked cutis
Phantom1	Caucasian	0.5	120	Epidermis
Phantom2	Caucasian	0.25	120	Epidermis
Phantom3	Caucasian	0.25	60	Epidermis
Phantom4	Caucasian	0.25	30	Epidermis
Phantom5	Caucasian	0.25	20	Epidermis
Phantom6	Caucasian	0.37	30	Epidermis
Phantom7	Caucasian	0.175	30	Dermis
Phantom8	Caucasian	0.08	30	Dermis
Phantom9	Negroid	0.5	240	Epidermis
Phantom10	Caucasian	0.3	80	Epidermis
Phantom11	Caucasian	0.4	80	Epidermis
Phantom12	Caucasian	0.05	120	Epidermis
